# Mechanistic Investigation into Crystallization of Hydrated Co-Amorphous Systems of Flurbiprofen and Lidocaine

**DOI:** 10.3390/pharmaceutics17020175

**Published:** 2025-01-30

**Authors:** Xiaoyue Xu, Holger Grohganz, Justyna Knapik-Kowalczuk, Marian Paluch, Thomas Rades

**Affiliations:** 1 Department of Pharmacy, University of Copenhagen, Universitetsparken 2, 2100 Copenhagen, Denmarkthomas.rades@sund.ku.dk (T.R.); 2 SMCEBI, Institute of Physics, Faculty of Science and Technology, University of Silesia in Katowice, 75 Pułku Piechoty 1a, 41-500 Chorzów, Poland

**Keywords:** co-amorphous, crystallization, thermodynamics, anti-plasticizing, effect of water, lidocaine

## Abstract

**Background:** It is generally accepted that water as a plasticizer can decrease the glass transition temperatures (T_g_s) of amorphous drugs and drug delivery systems, resulting in physical instabilities. However, a recent study has reported an anti-plasticizing effect of water on amorphous lidocaine (LID). In co-amorphous systems, LID might be used as a co-former to impair the plasticizing effect of water. **Method:** Flurbiprofen (FLB) was used to form a co-amorphous system with a mole fraction of LID of 0.8. The effect of water on the stability of co-amorphous FLB-LID upon hydration was investigated. The crystallization behaviors of anhydrous and hydrated co-amorphous FLB-LID systems were measured by an isothermal modulated differential scanning calorimetric (iMDSC) method. The relaxation times of the co-amorphous FLB-LID system upon hydration were measured by a broadband dielectric spectroscopy (BDS), and the differences in Gibbs free energy (ΔG) and entropy (ΔS) between the amorphous and crystalline phases were determined by differential scanning calorimetry (DSC). **Results:** It was found that the crystallization tendency of co-amorphous FLB-LID decreased with the addition of water. Molecular mobility and thermodynamic factors were both investigated to explain the difference in crystallization tendencies of co-amorphous FLB-LID upon hydration. **Conclusions:** The results of the study showed that LID could be used as an effective co-former to decrease the crystallization tendency of co-amorphous FLB-LID upon hydration by enhancing the entropic (ΔS) and thermodynamic activation barriers (TΔS)^3^/ΔG^2^) to crystallization.

## 1. Introduction

In the formulation of poorly water-soluble drugs, there is increasing interest in utilizing an amorphous form of the active pharmaceutical ingredient (API) rather than its crystalline counterpart due to the inherent dissolution rate enhancement and solubility improvement of the amorphous form [1]. However, the absence of crystalline lattice renders amorphous drugs and systems intrinsically unstable, resulting in crystallization [2]. Co-amorphous systems are thus developed as a formulation strategy to stabilize the amorphous API against crystallization through the use of a low molecular weight co-former, which could be either an inactive ingredient or another API [3,4].

The crystallization of amorphous drugs and co-amorphous systems is governed by nucleation and crystal growth, which are influenced by the combined effects of kinetics (such as molecular mobility) and thermodynamics (such as enthalpy, entropy, and Gibbs free energy) [5]. The global molecular mobility, usually referred to as α-relaxation, resulting from long-range cooperative motions of molecules, is responsible for the glass transition. It is generally accepted that enhanced global molecular mobility could kinetically promote the crystallization process of amorphous drugs [6,7]. In addition, the Johari–Goldstein (β) relaxation has been shown to have a strong correlation with the physical instability of amorphous drugs [7,8]. However, molecular mobility is not the only factor associated with the stability of amorphous drugs and drug formulations. Previous studies have highlighted the significance of thermodynamics in influencing the crystallization of amorphous drugs and co-amorphous systems. In 2022, Kumar and Suryanarayanan explained the observed differences in the stability of amorphous drugs in terms of the thermodynamic activation barriers for a range of drugs, including nifedipine, indomethacin, felodipine, and ketoconazole [9]. Furthermore, previous studies have identified the role of configurational entropy in stabilizing amorphous drugs and co-amorphous systems [10,11]. These findings suggest that both molecular mobility and thermodynamic factors play an important role in the crystallization process.

Previous observations on glassy materials indicate changes in mechanical properties, such as the modulus of a polymer at or near room temperature due to the addition of plasticizers and anti-plasticizers [12,13]. Specifically, the physical stability of amorphous drugs and co-amorphous systems is known to be sensitive to the addition of water due to its plasticizing effect [14,15]. The plasticizing effect of water on amorphous formulations can facilitate the crystallization process by decreasing their glass transition temperatures (T_g_s) and increasing molecular mobility [16]. The adsorption of water introduces excess free volume into amorphous drugs and co-amorphous systems, leading to a higher thermodynamic driving force for crystallization [14].

The term anti-plasticizer is used in this study to indicate a molecule with a lower T_g_ than the drug that nonetheless increases the T_g_ of that drug when being a part of the amorphous system [17]. Although water is generally observed to act as a plasticizer in amorphous pharmaceuticals, it has been shown to have an anti-plasticizing effect on certain drugs and polymers [17,18,19]. The mechanism of the anti-plasticizing effect of water is still under investigation and is being studied based on the structural properties of amorphous materials [20,21]. Some recent studies have found that water could also have an anti-plasticizing effect on some amorphous drugs, including prilocaine (PRL) and lidocaine (LID), resulting in increased T_g_s of PRL and LID upon hydration [17,18]. The T_g_s of hydrated PRL and LID exhibited maximum increases in the T_g_s of 4.1 K and 0.9 K, respectively, in comparison to their anhydrous counterparts [18]. The anti-plasticizing effect of water was found to be associated with the hydrogen bonding pattern between water and the carbonyl groups of PRL and LID [17,18,22,23]. Furthermore, previous work indicated that water could maintain an anti-plasticizing effect on co-amorphous systems of PRL and LID at various mole fractions of PRL [18]. These findings suggest that PRL and LID could be used as potential co-formers to impair the plasticizing effect of water in other co-amorphous systems. It was found that the effect of water on co-amorphous systems of nicotinamide (NIC) and PRL was correlated with the mole fractions of PRL. The anti-plasticizing effect of water could still be maintained at mole fractions of PRL of 0.8 and above, with increased T_g_s and reduced mobility upon hydration [24].

FLB was used as a model drug in this study due to its co-formability with LID [2]. Since the ΔpKa between FLB (pKa = 4.42) [25] and LID (pKa = 7.91) [26] exceeds three, proton transfer (i.e., ionic interactions) is likely [27]. A high degree of proton transfer between FLB and LID could alter the hydrogen bonding pattern between water and the co-former LID, leading to a plasticizing effect of water on the ionized LID. However, studies have shown that the molecular interactions between FLB and LID were primarily governed by hydrogen bonding (>99%) at a mole fraction of LID of 0.5 [28], facilitated by the tertiary amine group of LID [29]. Furthermore, only a limited degree of proton transfer in co-amorphous FLB-LID systems was observed with increasing LID fractions [30], consistent with findings in co-amorphous systems of naproxen and LID [31]. In addition, due to the fast crystallization of LID [32], it was only possible to obtain anhydrous co-amorphous FLB-LID systems with mole fractions of LID of 0.8 and below. The mole fraction of 0.8 was thus chosen to accommodate two effects. Firstly, to limit the degree of proton transfer, which has been shown to be limited at mole fractions of LID of 0.5 and above. Secondly, to reduce the likelihood for fast recrystallization of LID, observed for mole factions above 0.8. This mole fraction of LID was further used to investigate the effect of water on the co-amorphous FLB-LID system dominated by hydrogen bond formation.

It has been shown previously that the T_g_ of LID linearly increased upon hydration up to a water-to-drug molar ratio of X_H2O_ ≤ 50%, and the T_g_ of hydrated LID remained constant at X_H2O_ > 50% [18]. The aim of this study was to investigate the effect of water on the stability of the co-amorphous FLB-LID system at X_H2O_ ≤ 50%. Subsequent analyses on the T_g_ of co-amorphous FLB-LID were conducted at three hydration levels of X_H2O_ = 0%, 10%, and 50% to investigate if water is an anti-plasticizer for co-amorphous FLB-LID. The crystallization behaviors of anhydrous and hydrated co-amorphous FLB-LID systems were investigated using an isothermal modulated differential scanning calorimetric (iMDSC) method. This technique has been used in previous studies to describe the crystallization process of amorphous materials, particularly polymers [33,34,35], by determining the evolution of heat capacity during crystallization, providing a more accurate calculation of the crystalline fraction compared with the conventional DSC [36,37]. The time evolution of the crystallization fraction of co-amorphous FLB-LID upon hydration was investigated by the Johnson-Mehl-Avrami (JMA) equation. This model has been widely used to describe the crystallization behavior due to its simplicity and physically meaningful interpretation [38]. Furthermore, molecular mobility and thermodynamic factors were investigated to explain the difference in crystallization tendencies of the co-amorphous FLB-LID system upon hydration. The relaxation times of co-amorphous FLB-LID upon hydration were measured by a broadband dielectric spectroscopy (BDS), which has been widely used to determine the time scales of global and local motions related to dipole reorientation in amorphous materials [39,40]. In addition, the thermodynamics of crystallization were analyzed using configurational thermodynamics.

## 2. Materials and Methods

### 2.1. Materials

Flurbiprofen (FLB, MW = 244.26 g/mol) was purchased from Cayman Chemical (Ann Arbor, MI, USA). Lidocaine (LID, MW = 234.34 g/mol) was purchased from Sigma-Aldrich (St. Louis, MO, USA). Water (18.2 MΩ) was freshly prepared using a Milli-Q water system from ELGA LabWater (High Wycombe, UK). The chemical structures of FLB and LID are shown in Figure S1.

### 2.2. Methods

#### 2.2.1. Sample Preparation

Anhydrous and hydrated co-amorphous FLB-LID samples were prepared by melting crystalline mixtures of FLB and LID with a mole fraction of LID of 0.8 (i.e., LID/(FLB + LID)) and water, followed by quench-cooling. The crystalline mixtures of FLB and LID were obtained by gentle mechanical mixing using a plastic pin. Non-hermetically sealed pans were used for the anhydrous samples. For hydrated co-amorphous FLB-LID samples, a droplet of water was added to the anhydrous crystalline mixtures of FLB and LID with a mole fraction of LID of 0.8, followed by water evaporation monitored on a microbalance until the desired total mass was reached. This evaporation process allows precise control of the amount of water to reach the desired total sample mass [24]. Hermetically sealed pans were used for the hydrated samples. Our previous study suggests that the T_g_ of amorphous LID increased with the addition of water and reached a maximum at a water-to-drug molar ratio (i.e., water/(FLB + LID) × 100%) of X_H2O_ = 50% [18]. In the current study, water-to-drug molar ratios of X_H2O_ = 10% and X_H2O_ = 50% were used in hydrated co-amorphous FLB-LID samples. These anhydrous and hydrated co-amorphous samples were subsequently used for thermal and dielectric analyses.

#### 2.2.2. X-ray Powder Diffraction (XRPD)

The solid-state properties of the samples were investigated with an X’Pert PRO diffractometer (PANalytical, Almelo, The Netherlands) using Cu Kα radiation (λ = 1.54187 Å) at 45 kV and 40 mA. The samples were measured at room temperature and scanned from 5° to 35° 2θ in reflection mode at a scanning speed of 0.067 2θ/min and a step size of 0.026° 2θ. The XRPD data were analyzed using X’Pert HighScore Plus (v2.2.4) software (PANalytical, Almelo, Netherlands).

#### 2.2.3. Differential Scanning Calorimetry (DSC)

The thermal behavior of the co-amorphous samples was measured using a Discovery DSC (TA instruments, New Castle, DE, USA). The instrument was calibrated for temperature using indium and for heat capacity using sapphire discs. Temperature-modulated DSC (MDSC) was conducted under a constant nitrogen flow rate of 50 mL/min. The crystalline mixtures of PRL, LID, and water (total weight about 8 mg) were heated at a rate of 2 K/min with a modulation of 0.212 K every 40 s until above the melting temperature (T_m_). Co-amorphous FLB-LID samples were obtained by cooling the melted drug to 203 K at the maximal instrumental cooling rate. Co-amorphous FLB-LID systems can be made fully amorphous, as indicated by the glass transition events observed during the cooling process. The T_g_ was determined by reheating the sample at 2 K/min with a modulation of 0.212 K every 40 s. The reversing heat capacities of the crystalline mixtures (C_p_ (crystalline)) and co-amorphous samples (C_p_ (amorphous)) were obtained by deconvolution of the total heat capacities. The heat capacity change (ΔC_p_) was obtained by subtraction of the C_p_ of the crystalline mixture from the C_p_ of the co-amorphous sample at identical temperatures, based on the equation ΔC_p_ = C_p_ (amorphous–C_p_ (crystalline) [11]. These obtained values of C_p_ and ΔC_p_ were further used to calculate the crystallization kinetics in subsequent iMDSC measurements of the anhydrous and hydrated co-amorphous FLB-LID systems. The T_g_ of the co-amorphous FLB-LID sample was taken at the midpoint of the change in C_p_. The C_p_ measurements were conducted using two independent samples.

The measurements of the T_g_s of anhydrous and hydrated co-amorphous FLB-LID systems were repeated by melting the crystalline mixtures of FLB and LID and crystalline drugs at 10 K/min, followed by cooling the melted drug to 203 K at the maximal instrumental cooling rate. The T_g_ was determined by reheating the sample at 2 K/min with a modulation of 0.212 K every 40 s. The T_g_ values of anhydrous and hydrated co-amorphous FLB-LID systems with a mole fraction of 0.8 were determined for three independent samples and are reported as mean ± standard deviation. The T_g_ values of amorphous FLB and co-amorphous FLB-LID systems with mole fractions of LID of 0, 0.1, 0.3, 0.5, and 0.7 were also determined using the same protocol (*n* = 1).

#### 2.2.4. Isothermal Crystallization

The iMDSC measurements were used in this study to calculate the crystallization kinetics from the amorphous forms. This method has been shown to be beneficial in monitoring the crystallization process compared to the conventional DSC measurements [36,41]. In the iMDSC measurements, the amorphous materials are held at defined temperatures with temperature modulation for extended periods, allowing the measurement of changes in heat capacity during isothermal crystallization as a function of time [33]. For very slow crystallization processes, when using a conventional DSC, it is challenging to detect the changes in heat flow during crystallization over extended timescales, rendering it unsuitable for calculating the crystallized fraction [37].

Anhydrous and hydrated co-amorphous FLB-LID samples were prepared in situ by heating the crystalline mixtures at a rate of 10 K/min until melting, then initially equilibrating the sample at 203 K for 2 min, followed by equilibration at three defined temperatures (251 K, 249 K, and 245 K). These temperatures were chosen to investigate the isothermal crystallization behavior of anhydrous and hydrated systems using iMDSC measurements to detect the full crystallization process. Below 245 K, the isothermal crystallization processes of anhydrous and hydrated co-amorphous FLB-LID systems are very slow, and changes in heat capacity during crystallization were hard to detect, as they were confounded with the baseline of the instrument. Above 251 K, the isothermal crystallization is too fast to detect the full crystallization process. Upon isothermal hold, changes in C_p_ as a function of time at the defined temperatures (251 K, 249 K, and 245 K) were determined with a modulation of 0.212 K every 40 s [36]. The isothermal hold of the co-amorphous samples led to crystallization, resulting in a decrease of C_p_. After the isothermal hold, the co-amorphous samples were equilibrated at 203 K and then reheated to 363 K. This process was conducted to detect potential thermal transitions, including the glass transition of the amorphous components and the melting of crystallized components. These measurements were conducted using two independent samples. The changes in C_p_ of anhydrous and hydrated co-amorphous FLB-LID samples upon isothermal hold as a function of time were used to calculate the crystallization fractions (α_cr_):

(1) $$\alpha_{\mathrm{cr}}=\frac{C_{p}(\mathrm{amorphous})\;-\;C_{p}\left(t\right)}{\Delta C_{p}}\times 100\%$$

where ΔC_p_ is the heat capacity change at the defined temperatures (251 K, 249 K, and 245 K) of samples in the amorphous and crystalline forms. C_p_ (amorphous) is the C_p_ of anhydrous and hydrated co-amorphous FLB-LID systems at the defined temperatures. C_p_(t) is the change of the C_p_ upon isothermal hold as a function of time. The values of α_cr_ of anhydrous and hydrated co-amorphous FLB-LID samples were assumed independent of the crystalline forms of FLB, LID, and water due to the similarity in C_p_ values among the different polymorphs [9].

The Johnson–Mehl–Avrami (JMA) model was used to describe the crystallization kinetics of anhydrous and hydrated co-amorphous FLB-LID systems, focusing on the combined effect of nucleation and crystal growth. Compared to other models that independently describe nucleation and crystal growth, the JMA model is widely used in pharmaceutical research [42]. The JMA model is given as follows [43,44,45]:

(2) $$\alpha_{\mathrm{cr}}=1-\exp[-{\left(\mathrm{kt}\right)}^{n}]$$

where t is the crystallization time, and α_cr_ is the crystallization fraction. n is the Avrami exponent, describing the nucleation mechanism and growth dimension of crystals. k is the crystallization rate, which is dependent on the rate of nucleation and crystal growth. The JMA fitting was conducted by plotting ln[−ln(1 − α_cr_)] against ln(t).

#### 2.2.5. Broadband Dielectric Spectroscopy (BDS)

Dielectric measurements of anhydrous and hydrated co-amorphous FLB-LID samples were conducted using an Alpha dielectric spectrometer (Novocontrol GmbH, Montabaur, Germany) over the frequency range from 10^−1^ to 10^6^ Hz. The anhydrous and hydrated crystalline mixtures of FLB, LID, and water (total weight approximately 100 mg) were melted in a preheated oven at 363 K for 2 min. The dielectric measurements of co-amorphous samples were performed immediately after quench-cooling of the melt to 178 K in a parallel-plate cell made of stainless steel (10 mm and 0.1 mm gap provided by silica spacer fibers). The dielectric loss spectra were recorded from 178 K to 250 K with a step size of 5 K from 178 K to 228 K and a step size of 2 K above 228 K. The measurements were conducted using two independent samples.

#### 2.2.6. Molecular Mobility

The dielectric loss spectra were used to determine α-relaxation times (τ_α_) of the anhydrous and hydrated co-amorphous systems. The dielectric behaviors of the co-amorphous samples were characterized by the complex relative permittivity, ε*(ω), across varying frequencies. The Havriliak–Negami (HN) function with an additional term describing the DC-conductivity contribution was used to fit the dielectric data [46]:

(3) $$\varepsilon *(\omega )=\varepsilon_{\infty }+\frac{\Delta \varepsilon }{{\left(1+{\left(i\omega \tau_{\mathrm{HN}}\right)}^{\alpha }\right)}^{\beta }}+\frac{\sigma_{\mathrm{dc}}}{\varepsilon_{0}i\omega }$$

where ε_∞_ is the high-frequency limit permittivity, ε_0_ is the free space permittivity, and σ_dc_ is the DC-conductivity. Δε is the dielectric strength, ω is equal to 2πf, τ_HN_ is the HN relaxation time, and α and β represent the symmetric and asymmetric broadening of the relaxation peak. From the fitting parameters in Equation (3), the values of τ_α_ of anhydrous and hydrated co-amorphous FLB-LID systems were obtained from Equation (4):

(4) $$\tau_{\alpha }=\tau_{\mathrm{HN}}+{[\sin(\frac{\pi \alpha }{2+2\beta })]}^{1/\alpha }{\left[\frac{\pi \alpha \beta }{2+2\beta }\right]}^{-1/\alpha }$$

The temperature evolutions of the α-relaxation times (τ_α_(T)) of anhydrous and hydrated co-amorphous FLB-LID systems were further fitted with the Vogel–Fulcher–Tammann (VFT) equation [47]:

(5) $$\tau_{\alpha }\left(T\right)=\tau_{\infty }\exp[\frac{{\mathrm{DT}}_{0}}{T-T_{0}}]$$

where τ_∞_, T_0_, and D are fitting parameters. τ_∞_ is the relaxation time of unrestricted material, which should be in the order of around 10^−14^ s (i.e., vibrational frequency of molecules). T_0_ is the zero-mobility temperature, and D is the strength parameter.

## 3. Results and Discussions

### 3.1. T_g_s of Anhydrous and Hydrated Co-Amorphous FLB-LID Systems

**Figure 1:**
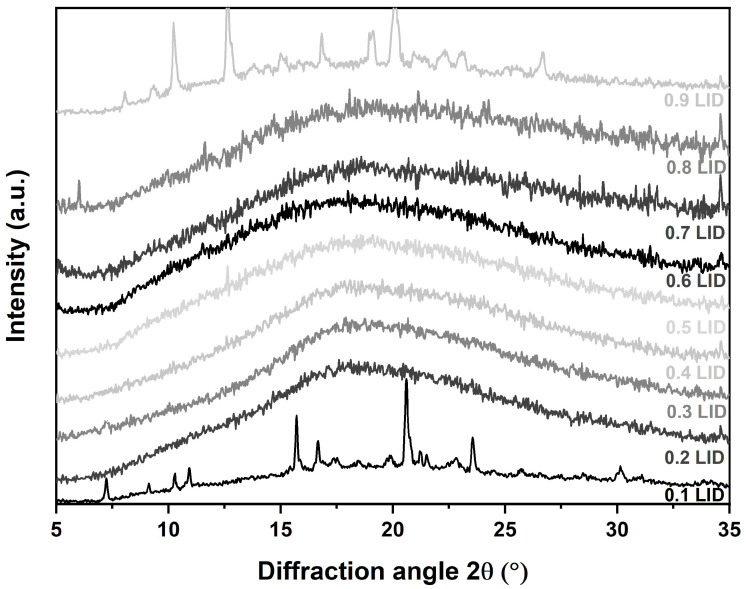
XRPD diffractograms of anhydrous co-amorphous FLB-LID systems with mole fractions of LID from 0.1 to 0.9.

As a first step, the establishment of co-amorphous systems with varying FLB-LID ratios was confirmed by XRPD (Figure 1). It can be seen that anhydrous co-amorphous FLB-LID with mole fractions of LID from 0.2 to 0.8 can be made amorphous at room temperature. Although these ambient temperature measurements cannot be directly correlated with the later DSC and BDS experiments, which were conducted at very low temperatures, the formation of co-amorphous systems was confirmed.

In the following, the influence of water on the T_g_ of co-amorphous FLB-LID systems was investigated. The experimental T_g_s of anhydrous and hydrated pure amorphous FLB and co-amorphous FLB-LID systems with mole fractions of LID of 0, 0.1, 0.3, 0.5, 0.7, and 0.8 are shown in Table S1. The changes in the experimental T_g_s of co-amorphous FLB-LID systems with high mole fractions of LID (0.7 and 0.8) upon hydration were lower than those with low mole fractions, indicating that the plasticizing effect of water on co-amorphous FLB-LID systems is more pronounced at lower mole fractions of LID.

The experimental T_g_ of the anhydrous co-amorphous FLB-LID system with a mole fraction of LID of 0.8 was found to be 228.6 ± 0.2 K. The experimental T_g_s of hydrated co-amorphous FLB-LID systems with water-to-drug molar ratios of X_H2O_ = 10% and X_H2O_ = 50% were found to be 227.8 ± 0.2 K, and 227.1 ± 1.0 K, respectively. The experimental T_g_ of the hydrated co-amorphous FLB-LID system with X_H2O_ = 10% exhibited a minimal reduction of 0.8 ± 0.2 K, compared with the T_g_ of the anhydrous system. Furthermore, there is no significant difference between the T_g_s of the anhydrous co-amorphous FLB-LID system and hydrated co-amorphous FLB-LID system with X_H2O_ = 50% (Student’s *t*-test, *p* < 0.05). Thus, it has been shown that the addition of water of X_H2O_ = 10% and X_H2O_ = 50% showed a minimal impact on the experimental T_g_ of the co-amorphous FLB-LID system with a mole fraction of LID of 0.8.

The effect of water on the T_g_ of the co-amorphous FLB-LID system was further fitted using the extended version of the Fox equation [48]:

(6) $$\frac{1}{T_{g123}}=\frac{w_{1}}{T_{g1}}+\frac{w_{2}}{T_{g2}}+\frac{w_{3}}{T_{g3}}$$

where T_g123_ is the theoretical T_g_ of anhydrous and hydrated co-amorphous FLB-LID systems, respectively. T_g1_, T_g2_, T_g3_, w_1_, w_2_, and w_3_ are the T_g_s and weight fractions of the individual components, respectively. For the first approach, considering water as a pure plasticizer, FLB (T_g1_ = 268.0 K), LID (T_g2_ = 209.8 K), and water (T_g3_ = 136.0 K) were treated as three individual components in Equation (6). Consequently, the theoretical T_g_ of the co-amorphous FLB-LID system was expected to decrease by 1.0 K with X_H2O_ = 10% and by 4.9 K with X_H2O_ = 50%, respectively. Inconsistencies were thus noted between the observed changes in the theoretical T_g_ of the co-amorphous FLB-LID system upon hydration and the experimental T_g_. In the second approach, water was considered a plasticizer for FLB and an anti-plasticizer for LID. It was assumed that water was distributed molecularly evenly between FLB and LID. The theoretical T_g_ of hydrated FLB, influenced by water as a plasticizer, was regarded as an individual component in Equation (6) (T_g1_ = 266.1 K for X_H2O_ = 10%, and 259.1 K for X_H2O_ = 50%). It has been previously reported that the T_g_ of hydrated LID exhibits a linear increase with the addition of water, reaching a maximum at X_H2O_ = 50% with an increase of 0.9 K (T_g2_ = 210.0 K for X_H2O_ = 10% and 210.9 K for X_H2O_ = 50%) [18]. Based on the second approach, the addition of water resulted in minimal changes in the theoretical T_g_ of the co-amorphous FLB-LID system, decreasing by 0.1 K for X_H2O_ = 10% and increasing by 0.4 K for X_H2O_ = 50%. Thus, the second approach leads to a better fit with the observed changes in the experimental T_g_ of the co-amorphous FLB-LID system upon hydration, compared with the first approach. Therefore, the minimal changes of the T_g_ of the co-amorphous FLB-LID system upon hydration are attributed to the dual effects of water, i.e., its plasticizing effect on FLB and its anti-plasticizing effect on LID.

### 3.2. Isothermal Crystallization

#### 3.2.1. Kinetics of Crystallization

**Figure 2:**
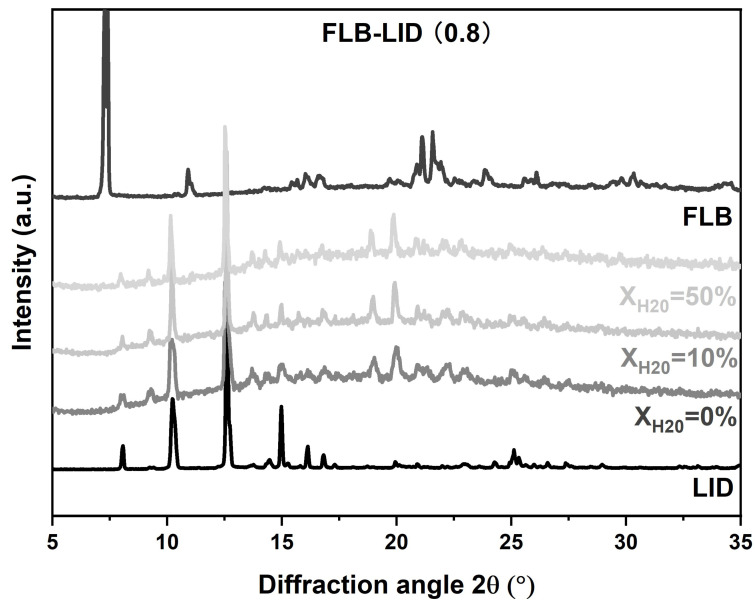
XRPD diffractograms of co-amorphous FLB-LID with X_H2O_ = 0%, 10%, 50% after isothermal partial crystallization, crystalline FLB, and crystalline LID.

The crystallization behavior of anhydrous and hydrated co-amorphous FLB-LID systems with a mole fraction of LID of 0.8 was investigated upon isothermal hold at three defined temperatures (245 K, 249 K, and 251 K). This temperature range above the T_g_ was chosen because the co-amorphous FLB-LID system exhibited a relatively high crystallization tendency compared to the behavior below the T_g_ [10]. XRPD on anhydrous and hydrated co-amorphous FLB-LID systems after isothermal partial crystallization showed reflections corresponding to LID at various water ratios, indicating that the crystallized component during isothermal crystallization mainly consisted of crystallized LID (Figure 2).

**Figure 3:**
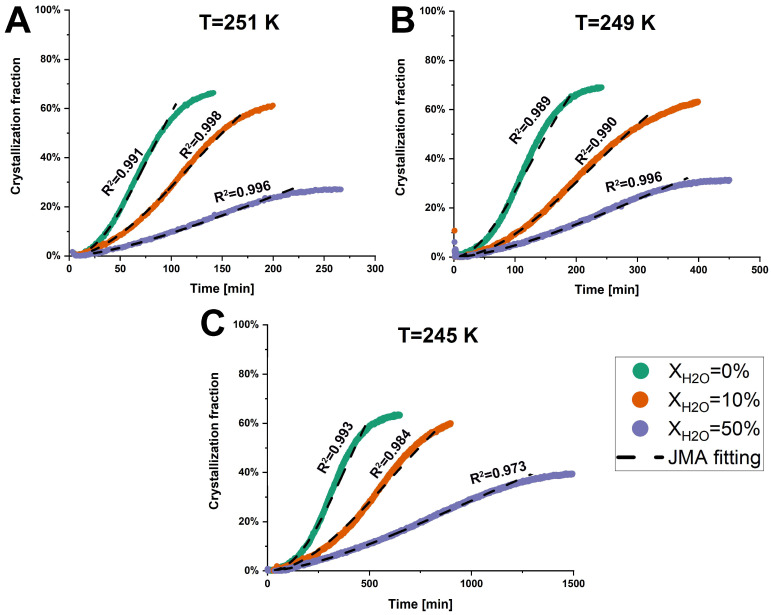
Crystallization fractions of anhydrous and hydrated co-amorphous FLB-LID systems with water-to-drug molar ratios of X_H2O_ = 0%, X_H2O_ = 10%, and X_H2O_ = 50%, as a function of time upon isothermal crystallization at 251 K (**A**), 249 K (**B**), and 245 K (**C**). The JMA fitting results are shown as black dashed lines.

The total heat flow of anhydrous and hydrated co-amorphous FLB-LID systems as a function of time, determined through the iMDSC measurements, is shown in Figure S2. Furthermore, the crystallization fraction of the co-amorphous FLB-LID system was calculated based on Equation (1). The anhydrous and hydrated co-amorphous FLB-LID systems began to crystallize at the onset of the isothermal hold at various temperatures (Figure 3). This fast crystallization might be attributed to the high concentration of LID within the co-amorphous FLB-LID system since the amorphous form of LID does not remain stable alone and crystallizes immediately [32]. Furthermore, the crystallization fractions and crystallization complete-time of anhydrous and hydrated co-amorphous FLB-LID systems at the defined temperatures are shown in Table 1. The crystallization fractions of the co-amorphous FLB-LID system at the various temperatures remained consistent, showing average crystallization fractions of 66.0 ± 2.5% for X_H2O_ = 0%, 60.9 ± 1.2% for X_H2O_ = 10%, and 32.6 ± 5.1% for X_H2O_ = 50%. The crystallization complete-time of anhydrous and hydrated co-amorphous FLB-LID systems decreased with the increasing temperatures. In contrast, at a constant temperature, the crystallization complete time of the co-amorphous FLB-LID system was increased upon hydration.

**Figure 4:**
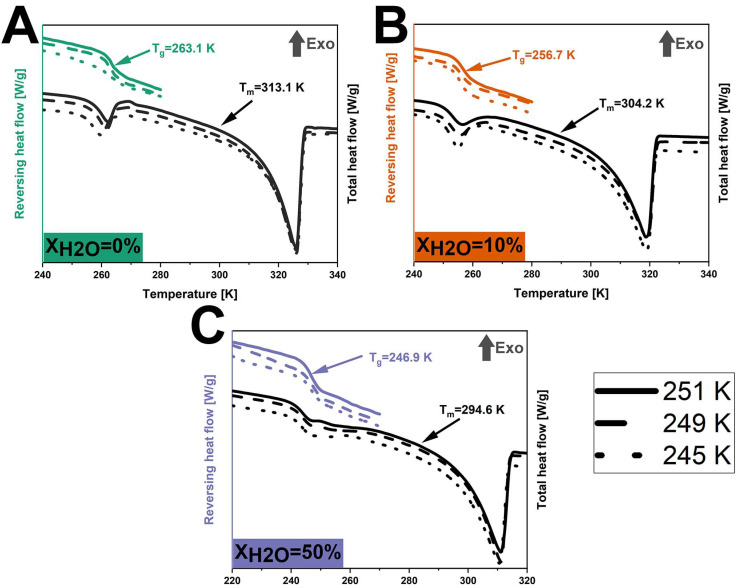
DSC thermograms of anhydrous and hydrated co-amorphous FLB-LID systems with water-to-drug molar ratios of X_H2O_ = 0% (**A**), X_H2O_ = 10% (**B**), and X_H2O_ = 50% (**C**) upon isothermal partial crystallization at 251 K (lines), 249 K (dashed lines), and 245 K (dots).

The JMA model was used to fit the crystallization fractions of anhydrous and hydrated co-amorphous FLB-LID systems, using Equation (2). The JMA model is based on the assumption that the growth of crystals occurs at a constant velocity [42]. It is assumed that only a single solid phase participates in the isothermal crystallization of anhydrous and hydrated systems, as indicated by a single melting endotherm attributed to the crystallized component, as shown in Figure 4. A detailed discussion follows in Section 3.2.2. For anhydrous and hydrated co-amorphous FLB-LID systems, the JMA model could be fitted to the isothermal crystallization process during the initial and middle stages of crystallization (Figure 3, black dashed lines). The JMA fitting parameters are shown in Table 1. However, in the final stages of crystallization of anhydrous and hydrated co-amorphous FLB-LID systems, the JMA model failed to fit the data. One possible reason could be related to the rapid growth of crystals occurring at the final stages of crystallization, which has been observed in other amorphous drugs and systems [49,50,51]. The Avrami exponent (n) of anhydrous and hydrated co-amorphous FLB-LID systems during isothermal crystallization was between 1.5 and 2.5, indicating small nucleation dimensions for the anhydrous and hydrated systems. The range of the n values of the co-amorphous FLB-LID system was consistent with the n values observed in a co-amorphous system of lurasidone hydrochloride and saccharin [10]. Compared with the anhydrous co-amorphous FLB-LID system, the values for the crystallization rate (k) of the co-amorphous FLB-LID system decreased upon hydration. This indicates that water decreased the rates of nucleation and crystal growth during isothermal crystallization.

Overall, considering the crystallization fractions, crystallization complete-time, and crystallization rates of anhydrous and hydrated co-amorphous FLB-LID systems, the crystallization tendency of the co-amorphous FLB-LID system was decreased upon hydration.

#### 3.2.2. Form of Water

The observed minimal changes in the T_g_s of co-amorphous FLB-LID systems upon hydration (Section 3.1) indicated that water interacted with both FLB and LID, with water showing an anti-plasticizing effect on LID and a plasticizing effect on FLB. Water in an amorphous matrix can be classified into three forms based on its thermodynamic properties: non-freezable water, freezable bound water, and free water [52]. Non-freezable water is closely bound to the amorphous matrix and does not exhibit a phase transition during calorimetric analysis. Freezable-bound water is less tightly bound to the matrix and shows melting and crystallization temperatures that are different from those of bulk water, whilst free water exhibits the thermodynamic properties of bulk water.

DSC was conducted to compare the thermal transitions between the anhydrous and hydrated systems after isothermal crystallization. After isothermal (partial) crystallization at 245 K, 249 K, and 251 K (Figure 4), all anhydrous co-amorphous FLB-LID systems showed a glass transition at 263.1 K. In a similar manner, at X_H2O_ = 10%, a T_g_ of 256.7 K was found for all three isothermal conditions, whilst at X_H2O_ = 50%, a T_g_ of 246.9 K was found for all three conditions. This consistency aligns with the similar crystallization fractions found at various temperatures, as shown in Table 1. The T_g_ at 263.1 K for the anhydrous co-amorphous FLB-LID system is closer to the T_g_ of pure FLB (268.0 K) than to the T_g_ of pure LID (209.8 K) (Figure 4A). This indicates that the remaining amorphous component mainly consisted of amorphous FLB and only a small fraction of amorphous LID. In addition, a single endothermic transition was observed at 313.1 K, attributed to the melting of the crystallized component. This T_m_ is closer to the T_m_ of LID (341.6 K) compared to the T_m_ of FLB (390.2 K), indicating that the crystallized component of the anhydrous system during isothermal crystallization mainly consisted of crystallized LID.

For hydrated co-amorphous systems with X_H2O_ = 10% and 50%, the T_g_ and T_m_ were lower than the anhydrous system, as shown in Figure 4. The decreased T_g_s of the hydrated co-amorphous FLB-LID systems could be attributed to the plasticizing effect of water on the remaining amorphous component since it mainly consists of amorphous FLB. In addition, the observed decrease in the T_m_ of the crystallized component for the hydrated co-amorphous FLB-LID system can be attributed to the effect of water on the crystallized solids [53].

No additional thermal transitions that could be attributed to water were observed for the hydrated systems compared with the anhydrous system. This observation indicates that the form of water existing in the hydrated co-amorphous FLB-LID system can be attributed to non-freezable water. This result is consistent with our previous findings that the anti-plasticizing effect of water on LID is due to a specific hydrogen bonding pattern between water and the carbonyl group of LID [22]. Overall, the thermograms of anhydrous and hydrated co-amorphous FLB-LID systems upon isothermal crystallization show that water closely interacts with the co-amorphous FLB-LID system, thus affecting the crystallization process of both FLB and LID within the system.

### 3.3. Molecular Mobility

**Figure 5:**
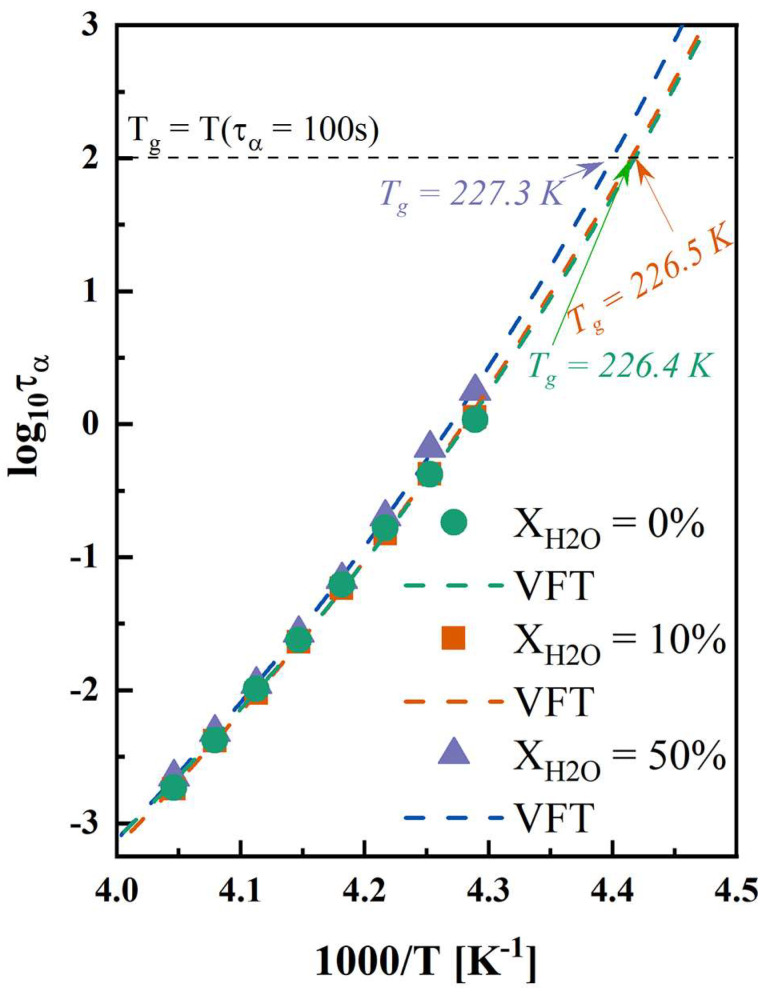
Temperature dependence of α-relaxation times of anhydrous and hydrated co-amorphous FLB-LID systems with water-to-drug molar ratios of X_H2O_ = 0% (green), X_H2O_ = 10% (orange), and X_H2O_ = 50% (purple). The VFT fitting results are shown as dashed lines in the corresponding colors.

For amorphous drugs and systems, isothermal hold above T_g_ can facilitate the crystallization process, attributed to the increased α-relaxation compared with the conditions below T_g_ [54]. Figure S3 shows the dielectric loss spectra of anhydrous and hydrated co-amorphous FLB-LID systems. As shown in Figure 3, the α-relaxation times of anhydrous and hydrated co-amorphous FLB-LID systems exhibited similar temperature dependences. The VFT fit (Equation (5)) was used to describe the temperature evolution of the α-relaxation times of anhydrous and hydrated co-amorphous FLB-LID systems (Figure 5, dashed lines). The VFT fitting parameters are shown in Table S2. The T_g_s, as determined by the VFT fit, were recognized at the point where τ_α_ reached 100 s. The T_g_s of anhydrous and hydrated co-amorphous FLB-LID systems were found to be 226.4 K for X_H2O_ = 0%, 226.5 K for X_H2O_ = 10%, and 227.3 K for X_H2O_ = 50%, in agreement with the T_g_s obtained from the DSC measurements. It has been shown that as the temperature increases, the molecular mobility of anhydrous and hydrated co-amorphous FLB-LID systems increases, as indicated by reduced α-relaxation times (τ_α_), corresponding to an increased crystallization complete-time. However, anhydrous and hydrated co-amorphous FLB-LID systems exhibited similar mobility at constant temperatures. Overall, it can be stated that molecular mobility alone cannot explain the isothermal crystallization behavior of the co-amorphous FLB-LID system during hydration.

### 3.4. Configurational Thermodynamics

In addition to molecular mobility, it has been previously demonstrated that configurational thermodynamics, particularly the differences in entropy and Gibbs free energy between the amorphous and crystalline phases, could influence the physical stability of amorphous drugs and systems [10,11]. Previous research has recognized that the difference in Gibbs free energy (ΔG) between the amorphous and crystalline phases is the thermodynamic driving force for crystallization [55,56]. However, in the case of over ten amorphous compounds, a greater entropy difference (ΔS) between the amorphous and crystalline phases indicated enhanced stability, regardless of the thermodynamic driving force involved [5,11]. Furthermore, for amorphous nifedipine, indomethacin, felodipine, and ketoconazole, the difference in crystallization propensity was explained in terms of the difference in the thermodynamic activation barrier, which correlated to the empirical relation ((TΔS)^3^/ΔG^2^) [9].

The ΔG of anhydrous and hydrated co-amorphous FLB-LID systems at a temperature (T) is related to the configurational enthalpy (ΔH) and entropy (ΔS) as shown in Equation (7):

(7) ΔG=ΔH−TΔS

The difference in C_p_s as a function of temperature (ΔC_p_(T)) between the amorphous and crystalline phases of the co-amorphous FLB-LID systems, as shown in Figure S4, was used to obtain the values of ΔH and ΔS, according to the following equations:

(8) $$\Delta H=\Delta H_{m}+\int_{Tm}^{T}\Delta Cp(T)dT$$

(9) $$\Delta S=\Delta S_{m}+\int_{Tm}^{T}\frac{\Delta Cp(T)}{T}dT$$

where ΔH_m_ and ΔS_m_ are the enthalpy and entropy of fusion for the fully crystallized component from the co-amorphous FLB-LID system, calculated by:

(10) $$\Delta H_{m}=\frac{\Delta H_{m}\left(\mathrm{cr}\right)}{\alpha_{\mathrm{cr}}}$$

(11) $$\Delta S_{m}=\frac{\Delta H_{m}}{T_{m}}$$

where T_m_ and ΔH_m_(cr) are the melting temperature and enthalpy of the crystalline component following isothermal crystallization, as determined by a DSC (Figure 4). α_cr_ is the average crystallization fraction of anhydrous and hydrated co-amorphous FLB-LID systems.

**Figure 6:**
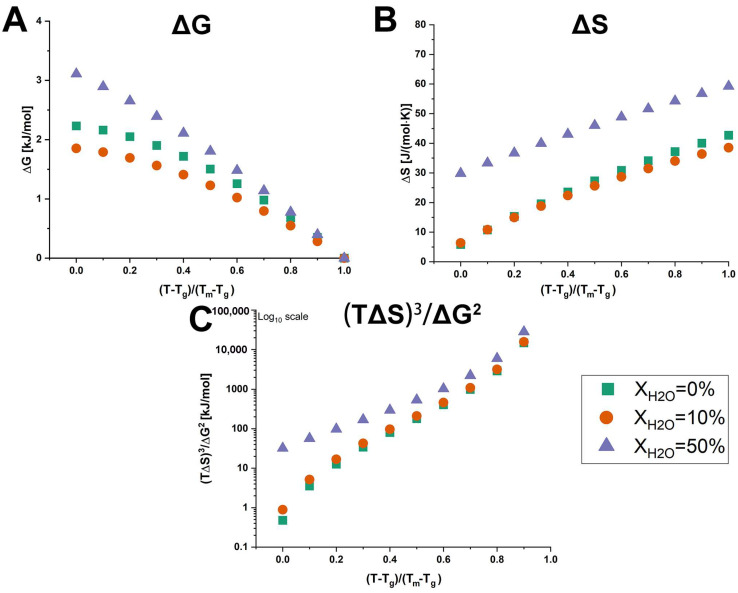
Temperature dependence of ΔG (**A**), ΔS (**B**), and (TΔS)^3^/ΔG^2^ (**C**) of anhydrous and hydrated co-amorphous FLB-LID systems with water-to-drug molar ratios of X_H2O_ = 0% (green), X_H2O_ = 10% (orange), and X_H2O_ = 50% (purple).

The calculated values for ΔG, ΔS, and (TΔS)^3^/ΔG^2^ of anhydrous and hydrated co-amorphous FLB-LID systems are shown in Figure 6. To compare the anhydrous and hydrated co-amorphous FLB-LID systems, which exhibited similar glass transitions but different melting points, the temperature scale was normalized using a reduced temperature, i.e., (T – T_g_)/(T_m_ – T_g_). The values of ΔG of anhydrous and hydrated co-amorphous FLB-LID systems decreased with increasing temperature, approaching zero at the melting point, consistent with previous findings [9,11]. Over the temperature range, the values of ΔG of anhydrous and hydrated co-amorphous FLB-LID systems were ranked as X_H2O_ = 50% > X_H2O_ = 0% > X_H2O_ = 10%. The higher value of ΔG indicates that the hydrated co-amorphous FLB-LID system with X_H2O_ = 50% exhibited a higher thermodynamic driving force towards crystallization compared with the other systems. This finding is in contrast to the decreased crystallization tendency of the co-amorphous FLB-LID system upon hydration, indicating that their crystallization tendencies cannot be explained solely by the value of ΔG. It was found that over most of the temperature range, the values of ΔS and (TΔS)^3^/ΔG^2^ of anhydrous and hydrated co-amorphous FLB-LID systems could be ranked as X_H2O_ = 50% > X_H2O_ = 10% ≈ X_H2O_ = 0%, which was consistent with the actual crystallization tendencies of these systems. Overall, the configurational thermodynamic analysis indicated that the decreased crystallization tendency of the hydrated co-amorphous FLB-LID system could be associated with the larger entropic (ΔS) and thermodynamic activation barriers ((TΔS)^3^/ΔG^2^) upon hydration.

## 4. Conclusions

In this study, the effect of LID as a co-former on the stability of the co-amorphous FLB-LID system, with a mole fraction of LID of 0.8 upon hydration, was investigated. In the co-amorphous FLB-LID system, water acted as a plasticizer for FLB, while it exhibited an anti-plasticizing effect on LID. The crystallization tendency of the co-amorphous FLB-LID system decreased with the addition of water. It was found that molecular mobility was not a dominating factor in stabilizing the co-amorphous FLB-LID system upon hydration. Rather, from the thermodynamic measurements, it was found that the hydrated co-amorphous FLB-LID system exhibited higher values for ΔS and (TΔS)^3^/ΔG^2^, compared with the anhydrous system. Therefore, the decrease in crystallization tendency of the co-amorphous FLB-LID system upon hydration was associated with higher entropic and thermodynamic activation barriers towards crystallization.

## Figures and Tables

**Table 1:** Crystallization fractions, complete-time, and JMA fitting parameters of anhydrous and hydrated co-amorphous FLB-LID systems with X_H2O_ = 0%, X_H2O_ = 10%, and X_H2O_ = 50%.

Temperature	X_H2O_	Crystallization Fraction	Crystallization Complete-Time [min]	n	k [min^−1^]
251 K	0%	66.1%	136.7	2.11	0.0094
	10%	60.1%	198.8	1.83	0.0055
	50%	27.1%	256.3	1.51	0.0021
249 K	0%	69.0%	237.7	1.92	0.0054
	10%	62.6%	389.3	1.87	0.0029
	50%	31.3%	447.2	1.54	0.0014
245 K	0%	62.9%	620.7	2.22	0.0020
	10%	60.0%	897.0	1.88	0.0011
	50%	39.3%	1476.2	1.54	0.0005

## Data Availability

The data presented in this study are available on request from the corresponding author.

## Supplementary Materials

The following supporting information can be downloaded at: https://www.mdpi.com/article/10.3390/pharmaceutics17020175/s1, Table S1: Experimental T_g_s (*n* = 1) of anhydrous and hydrated pure amorphous FLB and co-amorphous FLB-LID systems with mole fractions of LID of 0, 0.1, 0.3, 0.5, 0.7 0.8; Table S2: VFT fitting parameters of anhydrous and hydrated co-amorphous FLB-LID systems with water-to-drug molar ratios of X_H2O_ = 0%, X_H2O_ = 10%, and X_H2O_ = 50%; Figure S1: Chemical structures of FLB and LID; Figure S2: Total heat flow of anhydrous and hydrated co-amorphous FLB-LID systems with water-to-drug molar ratios of X_H2O_ = 0%, X_H2O_ = 10%, and X_H2O_ = 50% at 251 K, 249 K, and 245 K from iMDSC measurements; Figure S3: Dielectric loss spectra collected above the T_g_s of anhydrous and hydrated co-amorphous FLB-LID systems with water-to-drug molar ratios of X_H2O_ = 0%, X_H2O_ = 10%, and X_H2O_ = 50%; Figure S4: Difference in C_p_s as a function of temperature (ΔC_p_(T)) between the amorphous and crystalline phases of the anhydrous and hydrated co-amorphous FLB-LID systems with water-to-drug molar ratios of X_H2O_ = 0%, X_H2O_ = 10%, and X_H2O_ = 50%.

## References

[1] Hancock B.C., Zografi G. (1997). Characteristics and significance of the amorphous state in pharmaceutical systems. J. Pharm. Sci..

[2] Kasten G., Löbmann K., Grohganz H., Rades T. (2019). Co-former selection for co-amorphous drug-amino acid formulations. Int. J. Pharm..

[3] Dengale S.J., Grohganz H., Rades T., Löbmann K. (2016). Recent advances in co-amorphous drug formulations. Adv. Drug Deliv. Rev..

[4] Löbmann K., Laitinen R., Grohganz H., Gordon K.C., Strachan C., Rades T. (2011). Coamorphous drug systems: Enhanced physical stability and dissolution rate of indomethacin and naproxen. Mol. Pharm..

[5] Zhou D., Zhang G.G., Law D., Grant D.J., Schmitt E.A. (2002). Physical stability of amorphous pharmaceuticals: Importance of configurational thermodynamic quantities and molecular mobility. J. Pharm. Sci..

[6] Gupta P., Chawla G., Bansal A.K. (2004). Physical stability and solubility advantage from amorphous celecoxib: The role of thermodynamic quantities and molecular mobility. Mol. Pharm..

[7] Mehta M., Ragoonanan V., McKenna G.B., Suryanarayanan R. (2016). Correlation between Molecular Mobility and Physical Stability in Pharmaceutical Glasses. Mol. Pharm..

[8] Kissi E.O., Grohganz H., Löbmann K., Ruggiero M.T., Zeitler J.A., Rades T. (2018). Glass-transition temperature of the β-Relaxation as the major predictive parameter for recrystallization of neat amorphous drugs. J. Phys. Chem. B.

[9] Krishna Kumar N.S., Suryanarayanan R. (2022). Crystallization propensity of amorphous pharmaceuticals: Kinetics and thermodynamics. Mol. Pharm..

[10] Heng W., Song Y., Luo M., Hu E., Wei Y., Gao Y., Pang Z., Zhang J., Qian S. (2023). Mechanistic insights into the crystallization of coamorphous drug systems. J. Control Release.

[11] Graeser K.A., Patterson J.E., Zeitler J.A., Rades T. (2010). The role of configurational entropy in amorphous systems. Pharmaceutics.

[12] Chuang L., Panyoyai N., Katopo L., Shanks R., Kasapis S. (2016). Calcium chloride effects on the glass transition of condensed systems of potato starch. Food Chem..

[13] Mascia L., Kouparitsas Y., Nocita D., Bao X. (2020). Antiplasticization of polymer materials: Structural aspects and effects on mechanical and diffusion-controlled properties. Polymers.

[14] Newman A., Zografi G. (2019). An Examination of Water Vapor Sorption by Multicomponent Crystalline and Amorphous Solids and Its Effects on Their Solid-State Properties. J. Pharm. Sci..

[15] Hancock B.C., Zografi G. (1994). The relationship between the glass transition temperature and the water content of amorphous pharmaceutical solids. Pharm. Res..

[16] Heljo V.P., Nordberg A., Tenho M., Virtanen T., Jouppila K., Salonen J., Maunu S.L., Juppo A.M. (2012). The effect of water plasticization on the molecular mobility and crystallization tendency of amorphous disaccharides. Pharm. Res..

[17] Ruiz G.N., Romanini M.A.-O.X., Hauptmann A., Loerting T., Shalaev E., Tamarit J.A.-O., Pardo L.A.-O., Macovez R.A.-O. (2017). Genuine antiplasticizing effect of water on a glass-former drug. Sci. Rep..

[18] Xu X., Grohganz H., Rades T. (2022). Influence of water on amorphous lidocaine. Mol. Pharm..

[19] Chen Y., Tang T., Ayranci C. (2022). Moisture-induced anti-plasticization of polylactic acid: Experiments and modeling. J. Appl. Polym. Sci..

[20] Wang J.L., Cheng F., Zhu P.X. (2014). Structure and properties of urea-plasticized starch films with different urea contents. Carbohydr. Polym..

[21] Lerbret A., Affouard F. (2017). Molecular Packing, Hydrogen bonding, and fast dynamics in lysozyme/trehalose/glycerol and trehalose/glycerol glasses at low hydration. J. Phys. Chem. B.

[22] Xu X., Rades T., Grohganz H. (2024). Molecular interactions of hydrated co-amorphous systems of prilocaine and lidocaine. Int. J. Pharm..

[23] Xu X., Grohganz H., Rades T. (2024). Anti-plasticizing effect of water on prilocaine and lidocaine—The role of the hydrogen bonding pattern. Phys. Chem. Chem. Phys..

[24] Xu X., Rades T., Grohganz H. (2023). Thermal investigation on hydrated co-amorphous systems of nicotinamide and prilocaine. Eur. J. Pharm. Biopharm..

[25] Flurbiprofen. SCIFINDER. American Chemical Society. n.d. (CAS RN: 5104-49-4). https://scifinder-n.cas.org/searchDetail/substance/66ab53ab990d45148c8b114f/substanceDetails.

[26] Lidocaine. SCIFINDER. American Chemical Society. n.d. (CAS RN: 137-58-6). https://scifinder-n.cas.org/searchDetail/substance/66ab5441990d45148c8b1f9d/substanceDetails.

[27] Childs S.L., Stahly G.P., Park A. (2007). The salt-cocrystal continuum: The influence of crystal structure on ionization state. Mol. Pharm..

[28] Moreira D.N., Fresno N., Pérez-Fernández R., Frizzo C.P., Goya P., Marco C., Martins M.A.P., Elguero J. (2015). Brønsted acid–base pairs of drugs as dual ionic liquids: NMR ionicity studies. Tetrahedron.

[29] Wang H., Gurau G., Shamshina J., Cojocaru O.A., Janikowski J., MacFarlane D.R., Davis J.H., Rogers R.D. (2014). Simultaneous membrane transport of two active pharmaceutical ingredients by charge assisted hydrogen bond complex formation. Chem. Sci..

[30] Marei H.F., Arafa M.F., Essa E.A., El Maghraby G.M. (2021). Lidocaine as eutectic forming drug for enhanced transdermal delivery of nonsteroidal anti-inflammatory drugs. J. Drug Deliv. Sci. Technol..

[31] Fiandaca M., Dalwadi G., Wigent R., Gupta P. (2020). Ionic liquid formation with deep eutectic forces at an atypical ratio (2:1) of naproxen to lidocaine in the solid-state, thermal characterization and FTIR investigation. Int. J. Pharm..

[32] Blaabjerg L.I., Lindenberg E., Löbmann K., Grohganz H., Rades T. (2016). Glass Forming Ability of Amorphous Drugs Investigated by Continuous Cooling and Isothermal Transformation. Mol. Pharm..

[33] Toda A., Arita T., Tomita C., Hikosaka M. (1999). Temperature-modulated DSC applied to the transformation kinetics of polymer crystallization. Polym. J..

[34] Pak J., Wunderlich B. (2001). Melting and crystallization of polyethylene of different molar mass by calorimetry. Macromolecules.

[35] Righetti M.C., Prevosto D., Tombari E. (2016). Time and temperature evolution of the rigid amorphous fraction and differently constrained amorphous fractions in PLLA. Macromol. Chem. Phys..

[36] Righetti M.C. (2017). Crystallization of polymers investigated by temperature-modulated DSC. Materials.

[37] Otun S.O., Meehan E., Qi S., Craig D.Q. (2015). The use of quasi-isothermal modulated temperature differential scanning calorimetry for the characterization of slow crystallization processes in lipid-based solid self-emulsifying systems. Pharm. Res..

[38] Svoboda R. (2021). Crystallization of glasses—When to use the Johnson-Mehl-Avrami kinetics?. J. Eur. Ceram. Soc..

[39] Kramarczyk D., Knapik-Kowalczuk J., Smolka W., Monteiro M.F., Tajber L., Paluch M. (2022). Inhibition of celecoxib crystallization by mesoporous silica—Molecular dynamics studies leading to the discovery of the stabilization origin. Eur. J. Pharm. Sci..

[40] Knapik-Kowalczuk J., Rams-Baron M., Paluch M. (2021). Current research trends in dielectric relaxation studies of amorphous pharmaceuticals: Physical stability, tautomerism, and the role of hydrogen bonding. TrAC Trends Anal. Chem..

[41] Androsch R., Wunderlich B. (2003). Specific reversible melting of polymers. J. Polym. Sci. Part B Polym. Phys..

[42] Bhugra C., Pikal M.J. (2008). Role of thermodynamic, molecular, and kinetic factors in crystallization from the amorphous state. J. Pharm. Sci..

[43] Avrami M. (1939). Kinetics of Phase Change. I General Theory. J. Chem. Phys..

[44] Avrami M. (1940). Kinetics of Phase Change. II Transformation-Time Relations for Random Distribution of Nuclei. J. Chem. Phys..

[45] Avrami M. (1941). Granulation, Phase Change, and Microstructure Kinetics of Phase Change. III. J. Chem. Phys..

[46] Havriliak S., Negami S. (1967). A complex plane representation of dielectric and mechanical relaxation processes in some polymers. Polymer.

[47] Fulcher G.S. (1925). Analysis of recent measurements of the viscosity of glasses. J. Am. Ceram. Soc..

[48] Fox T.G., Flory P.J. (1950). Second-Order Transition Temperatures and Related Properties of Polystyrene. I. Influence of Molecular Weight. J. Appl. Phys..

[49] Cheng S., McKenna G.B. (2021). Isothermal crystallization and time-temperature transformation of amorphous nifedipine: A case of polymorphism formation and conversion. Mol. Pharm..

[50] Ruiz G.N., Romanini M., Barrio M., Tamarit J.L., Pardo L.C., Macovez R. (2017). Relaxation dynamics vs crystallization kinetics in the amorphous state: The case of stiripentol. Mol. Pharm..

[51] Madbouly S.A., Mansour A.A., Abdou N.Y. (2007). Crystallization kinetics of PHB/PVAc blends using time resolved dielectric spectroscopy. Eur. Polym. J..

[52] Pirayavaraporn C., Rades T., Gordon K.C., Tucker I.G. (2013). Quantification of the types of water in Eudragit RLPO polymer and the kinetics of water loss using FTIR. Int. J. Pharm..

[53] Inoue T. (1994). Effect of water on melting phase relations and melt composition in the system Mg_2_SiO_4_·MgSiO_3_·H_2_O up to 15 GPa. Phys. Earth Planet. Inter..

[54] Zhou D., Zhang G.G., Law D., Grant D.J., Schmitt E.A. (2008). Thermodynamics, molecular mobility and crystallization kinetics of amorphous griseofulvin. Mol. Pharm..

[55] Lauritzen J.I., Hoffman J.D. (1960). Theory of formation of polymer crystals with folded chains in dilute solution. J. Res. Natl. Bur. Stand. A Phys. Chem..

[56] Qian K.K., Bogner R.H. (2012). Application of mesoporous silicon dioxide and silicate in oral amorphous drug delivery systems. J. Pharm. Sci..

