# Supplementary material for: Mechanistic Investigation into Crystallization of Hydrated Co-Amorphous Systems of Flurbiprofen and Lidocaine
Source: Pharmaceutics. 2025 Jan 30;17(2):175. doi: 10.3390/pharmaceutics17020175 (PMC11859373; doi:10.3390/pharmaceutics17020175)
Supplement: Supplementary file 1 [file pharmaceutics-17-00175-s001.zip › pharmaceutics-3394245-supplementary.pdf]

Supporting information

Mechanistic Investigation into Crystallization of Hydrated Co-Amorphous Systems of Flurbiprofen and Lidocaine

Xiaoyue Xu [1], Holger Grohganz [1,*], Justyna Knapik-Kowalczuk [2], Marian Paluch [2] and Thomas Rades [1]

[1] Department of Pharmacy, University of Copenhagen, Universitetsparken 2, 2100 Copenhagen, Denmark
[2] SMCEBI, Institute of Physics, Faculty of Science and Technology, University of Silesia in Katowice, 75 Pułku Piechoty 1a, 41-500 Chorzów, Poland
[*] Correspondence: holger.grohganz@sund.ku.dk

Table S1. Experimental T_gs (n=1) of anhydrous and hydrated pure amorphous FLB and co-amorphous FLB-LID systems with mole fractions of LID of 0, 0.1, 0.3, 0.5, 0.7 0.8.

Mole fraction of LID	T_g [K] X_{H2O}=0%	T_g [K] X_{H2O}=10%	ΔT_g [K] T_g(10%)- T_g(0%)	T_g [K] X_{H2O}=50%	ΔT_g [K] T_g(50%)- T_g(0%)
0	268.0	258.7	-9.3	260.4	-7.6
0.1	273.6	263.1	-10.5	254.2	-19.44
0.3	283.0	275.7	-7.3	263.3	-19.7
0.5	264.7	260.9	-3.7	256.3	-8.4
0.7	240.7	239.7	-0.98	238.8	-1.97
0.8	228.8	227.6	-1.2	227.9	-0.9

Table S2. VFT fitting parameters of anhydrous and hydrated co-amorphous FLB-LID systems with water-to-drug molar ratios of X_{H2O}=0%, X_{H2O}=10%, and X_{H2O}=50%.

X_{H20}	$Log_{10}(\tau_\infty$ [s])	T_0 [K]	$D = BT_0$
0%	-14.0 ± 2.5	176.1 ± 1.1	1852 ± 31
10%	-14.8 ± 1.8	173.8 ± 0.4	2034 ± 12
50%	-14.3 ± 2.1	177.6 ± 9.9	1868 ± 61

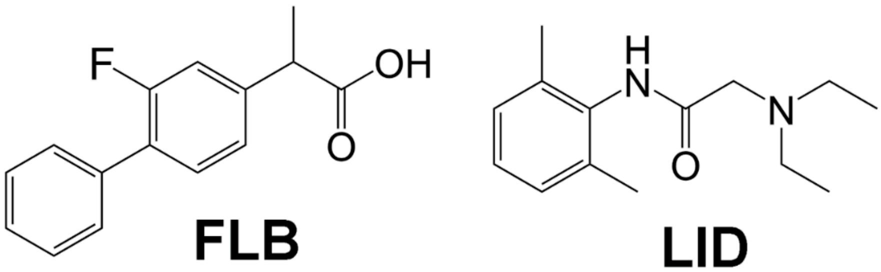

Figure S1. Chemical structures of FLB (A) and LID (B).

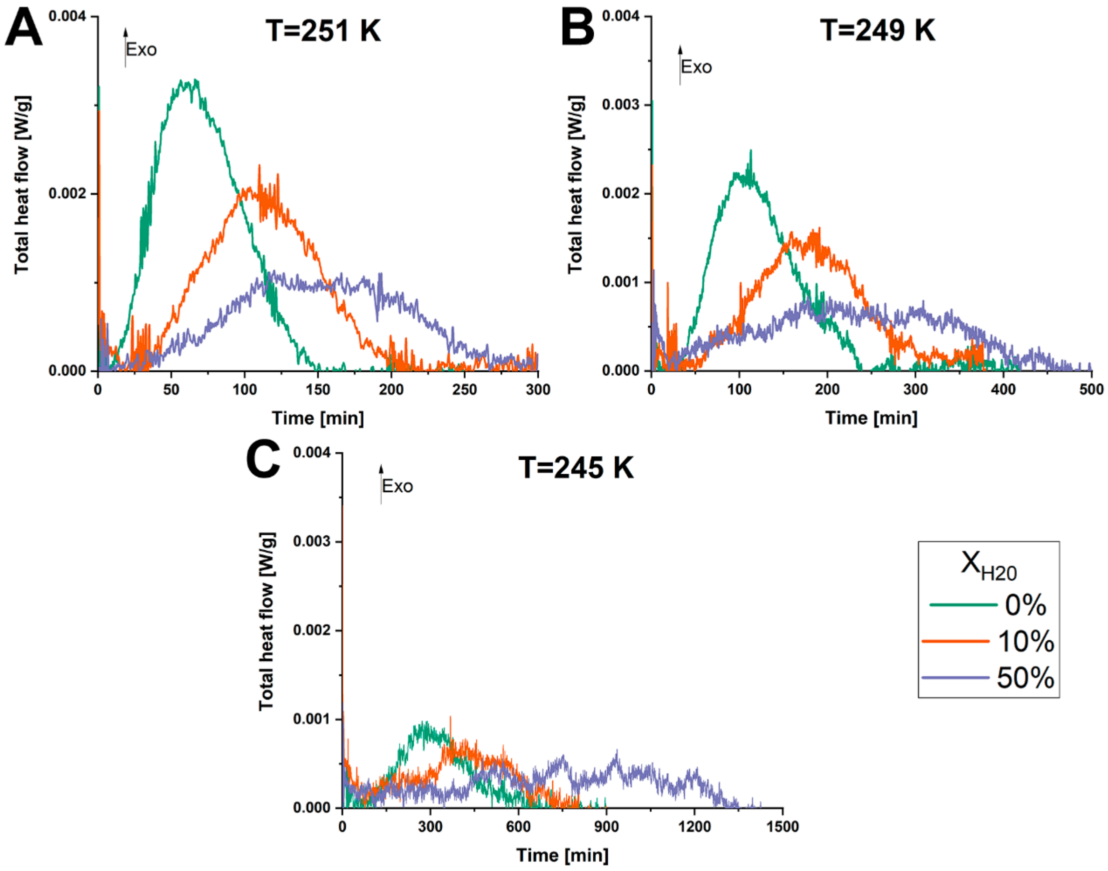

Figure S2. Total heat flow of anhydrous and hydrated co-amorphous FLB-LID systems with water-to-drug molar ratios of X_{H2O}=0% (green), X_{H2O}=10% (orange), and X_{H2O}=50% (purple) at 251 K (A), 249 K (B), and 245 K (C) from iMDSC measurements.

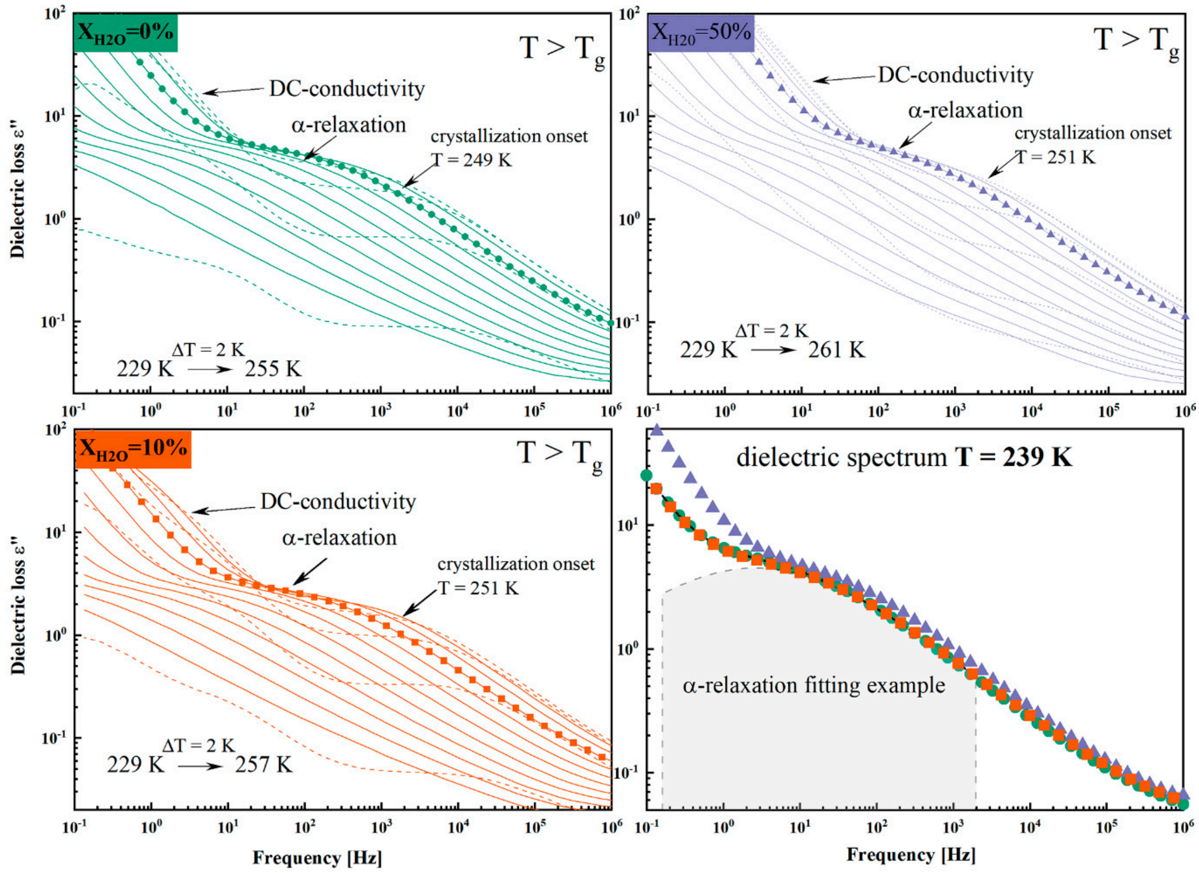

Figure S3. Dielectric loss spectra collected above the T_gs of anhydrous and hydrated co-amorphous FLB-LID systems with water-to-drug molar ratios of X_{H2O}=0% (green), X_{H2O}=10% (orange), and X_{H2O}=50% (purple).

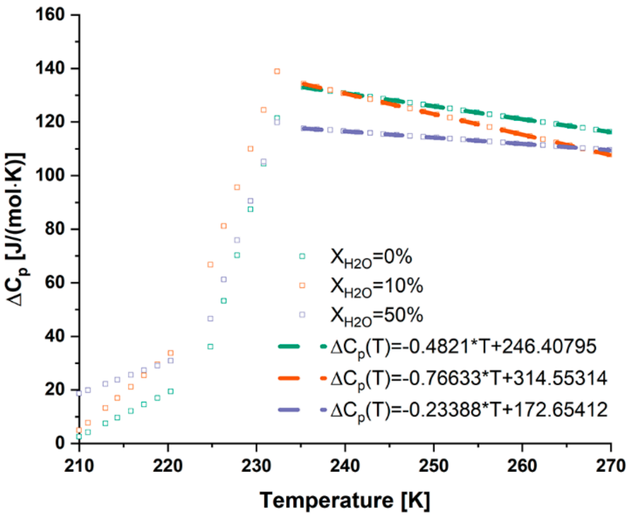

Figure S4. Difference in C_ps as a function of temperature ($\Delta C_p(T)$) between the amorphous and crystalline phases of the anhydrous and hydrated co-amorphous FLB-LID systems with water-to-drug molar ratios of X_{H2O}=0% (green), X_{H2O}=10% (orange), and X_{H2O}=50% (purple). The linear regression fit to the values of ΔC_p above the T_gs are shown in the dashed lines (R^2>0.990).
